# Hepatitis B Virus X Gene Differentially Modulates Subgenotype F1b and F4 Replication

**DOI:** 10.3390/v11070655

**Published:** 2019-07-18

**Authors:** María Mercedes Elizalde, Micaela Speroni, Rodolfo Héctor Campos, Diego Martín Flichman

**Affiliations:** 1 Cátedra de Virología, Departamento de Microbiología, Inmunología, Biotecnología y Genética, Facultad de Farmacia y Bioquímica, Universidad de Buenos Aires, Junín 956, Buenos Aires 1113, Argentina; 2 Consejo Nacional de Investigaciones Científicas y Técnicas (CONICET), Buenos Aires 1425, Argentina

**Keywords:** Hepatitis B virus, X gene, X protein, subgenotype F1b, subgenotype F4, differential replication

## Abstract

Hepatitis B virus (HBV) is classified into ten genotypes and numerous subgenotypes (sgt). In particular, sgt F1b and sgt F4, native of Latin America, have been associated with differences in clinical and virological characteristics. Hepatitis B virus X protein (HBx) is a multifunctional regulatory protein associated with the modulation of viral transcription and replication. In this work, we analyzed the role of the X gene and the encoded X protein in sgtF1b and sgtF4 replication. Transfection with HBx deficient genomes revealed remarkable differences in the replicative capacity of sgtF1b and sgtF4 mutants. The silencing of HBx increased sgtF1b X(-) transcription and replication by more than 2.5 fold compared to the wild type variant, while it decreased sgtF4 X(-) transcription and replication by more than 3 fold. Trans-complementation of HBx restore sgtF1b and sgtF4 wild type transcription and replication levels. In addition, transfection with chimeric variants, carrying wild type (F1b/XF4 and F4/XF1b) or mutated (F1b/X(-)F4 and F4/X(-)F1b) X gene of one sgt in the backbone of the other sgt, showed that the nucleotide sequence of the X gene, that includes regulatory elements that modulate pgRNA transcription, was responsible for the disparity observed between sgtF1b X(-) and sgtF4 X(-). These results showed that sgtF1b and sgtF4 X gene play a central role in regulating HBV transcription and replication, which eventually lead to a common purpose, to reach wild type replication levels of sgtF1b and sgtF4 viruses.

## 1. Introduction

Hepatitis B virus (HBV) is an important human pathogen with an estimated 257 million individuals chronically infected worldwide. HBV is a major etiological agent of severe liver diseases, including liver cirrhosis and hepatocellular carcinoma (HCC), which causes approximately 887.000 deaths annually [1].

HBV is a small, enveloped DNA virus that replicates via an RNA intermediate. The HBV genome consists of a partially double-stranded relaxed circular DNA (rcDNA) of 3.2 kb. Upon infection, host DNA enzymes repaired the rcDNA to generate a covalently closed circular DNA (cccDNA). The cccDNA serves as a transcriptional template for the different viral RNAs. The 3.5 kb pre-genomic RNA (pgRNA) is the mRNA for the synthesis of the polymerase and core proteins, and also the template for reverse transcription. The 3.5 kb precore RNA encodes HBV e antigen (HBeAg), the 2.4 kb and 2.1 kb transcripts generate the envelope proteins, whereas the 0.7 kb transcript encodes the X protein (HBx) [2].

HBx is a 154 amino acid protein with an N-terminal negative regulatory domain and a C-terminal transactivation domain. By interacting with transcription factors or signal transduction pathways, HBx affects several cellular functions such as calcium regulation, gene transcription, cell proliferation, DNA repair, apoptosis and autophagy [3,4,5]. Nevertheless, its exact role in HBV life cycle has not been clearly determined yet.

Although HBx is not essential for viral replication [6], recent reports have shown that HBx enhance HBV replication and transcription via regulation of viral transcription [6,7,8], and influencing the epigenetic control of cccDNA [9,10,11]. Whereby, HBx plays a critical, but not fully understood, role in HBV replication.

HBV has been classified into ten genotypes (named A to J) and numerous subgenotypes (sgt) [12,13]. The genotypes and sgts show different geographical distribution and have been associated with differences in clinical and virological characteristics, such as disease progression and prognosis [14,15]. The Asian and European genotypes (A to D) have been the most extensively studied; while, there is a paucity of data regarding other genotypes, including genotype F.

In South America, sgtF1b and sgtF4, are the most prevalent genotype F circulating sgts [16,17,18,19]. Recent studies have shown differences at the molecular and clinical level between these two sgts. In transfection and co-transfection assays, sgtF1b showed a higher replication capacity and a major fitness compared to sgtF4 [20,21]. In addition, individuals infected with sgtF1b seroconvert to anti-HBe later in life than those infected with sgtF4 [18]. Moreover, growing evidence has shown a close association of sgtF1b with a more severe course of chronic infections, and a high correlation to HCC progression exists among Alaska and Peru people [22,23,24,25].

The aim of this study was to address the in-deep characterization of the X gene and the encoded X protein of sgtF1b and sgtF4 in order to unravel their role in HBV replication.

## 2. Material and Methods

### 2.1. Cell Culture

Human hepatoma cell line HuH-7 (JCRB Cell Bank #0403) was cultured in Dulbecco’s modified Eagle’s medium (DMEM; GIBCO, Carlsbad, CA, USA) supplemented with 10% fetal bovine serum (Internegocios, Mercedes, Argentina), 1 mM nonessential amino acids (GIBCO, Carlsbad, CA, USA), 0.15% sodium bicarbonate, 100 UI/mL penicillin and 100 µg/mL streptomycin [26]. Cells were maintained at 37 °C in a humidified atmosphere containing 5% CO_2_.

### 2.2. Viral Variants

**Figure 1:**
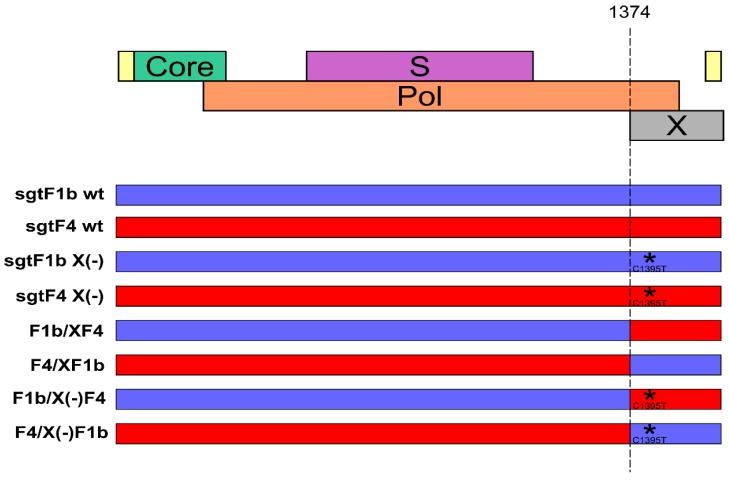
Schematic representation of the Hepatitis B virus (HBV) genomes used in the study. The asterisk represents the introduction of C1395T mutation that abolishes the expression of HBx.

Vector pUC19 containing full-length HBV genomes of sgtF1b and sgtF4 wild type (sgtF1b wt, sgtF4 wt), HBx minus (sgtF1b X(-), sgtF4 X(-)), HBx chimera wild type (F1b/XF4, F4/XF1b) and HBx minus chimera (F1b/X(-)F4 and F4/X(-)F1b) were analyzed in this study (Figure 1).

The generation of sgtF1b wt and sgtF4 wt plasmids was previously described [20]. Briefly, HBV DNA was extracted from serum samples of HBeAg positive patients chronically infected with HBV sgtF1b and sgtF4. Full-length HBV genomes were amplified according to the Günther’s method [27]. For efficient directional cloning of the PCR products, P1 sense primer was modified to contain the NdeI/BspQI sites: 5′CCGGACATATGAT**GCTCTTCT**TTTTCACCTCTGCCTAATCATC′3 (NdeI site is underlined and BspQI site is bold; NdeI site was inserted instead of HindIII because genotype F is cut by the latter) and P2 antisense primer was modified to contain the SacI/BspQI sites: 5′CCGGAGAGCTCAT**GCTCTTCA**AAAAGTTGCATGGTGCTGGTG′3 (SacI site is underlined and BspQI site is bold). The PCR was performed using the Expand high-fidelity PCR system (Roche, Mannheim, Germany) according to the manufacturer’s instructions. The amplified HBV DNA was digested with the NdeI and SacI restriction enzymes (New England Biolabs, Beverly, MA, USA) and separated by agarose gel electrophoresis. The 3.2 kb fragments were recovered by gel purification using the PureLink Quick Gel Extraction Kit (Invitrogen, Carlsbad, CA, USA) and inserted into NdeI/SacI sites of pUC19 vector to generate sgtF1b wt and sgtF4 wt plasmids.

To abolish the expression of the X protein, sgtF1b and sgtF4 X minus (-) genomes were designed by site-directed mutagenesis of sgtF1b wt and sgtF4 wt genomes via introduction of C1395T mutation. This mutation results in a stop codon in the X gene (codon 8) without affecting the polymerase gene product. Site-directed mutagenesis was carried out by PCR amplification of the sgtF1b wt and sgtF4 wt plasmids with primers: sense 5′ TCCTTCCCATGGCTGCTCGGWTGTGCTGC**T**AACTG 3′ (nt 1366 to 1400), including NcoI restriction site (underlined) and carrying the C to T mutation at nt 1395 (bold) and antisense 5′ CCGGAGAGCTCATGCTCTTCAAAAAGTTGCATGGTGCTGGTG 3′ (nt 1825 to 1804) including SacI restriction site (underlined). The amplification conditions were: an initial denaturation step at 94 °C for 5 min, then 94 °C for 30 s, 53 °C for 30 s, 72 °C for 30 s for 36 cycles and a final extension at 72 °C for 5 min. The mutated PCR products were digested with NcoI and SacI restriction enzymes (New England Biolabs, Beverly, MA, USA) and exchange with the corresponding fragment in the sgtF1b wt and sgtF4 wt plasmids (Figure 1). The genomes were sequenced in order to confirm the presence of the mutation in the X gene.

For the construction of HBV genomes with chimeric wild type (F1b/XF4 and F4/XF1b) and chimeric mutated X genes (F1b/X(-)F4 and F4/X(-)F1b), sgtF1b wt, sgtF4 wt and sgtF1b X(-), sgtF4 X(-) plasmids were digested with NcoI (nt 1372 to 1377) and SacI restriction enzymes (New England Biolabs, Beverly, MA, USA). The digested X gene products (XF1b, XF4 and XF1b(-), XF4(-)) were swapped into the other digested vector (F4, F1b and F4(-), F1b(-), respectively) (Figure 1). The genomes were sequenced in order to confirm the presence of the chimeric X genes.

The GenBank accession numbers for these constructs were: MK808033, MK808034, MK808035, MK808036, MK808037 and MK808038, respectively.

The pCH-9/3091 POL minus plasmid (kindly provided by Dr. Michael Nassal) was used as non-replicative control [28].

### 2.3. HBx Expression Plasmids

For trans-complementation assays, sgtF1b HBx and sgtF4 HBx expression plasmids were constructed from the previously described HBV full-length sgtF1 wt and sgtF4 wt plasmids.

The X gene was amplified by PCR using the following primers: sense 5′ AGCCACCATGGCTGCTCGGTTGTGC 3′ (nt 1368 to 1392) and antisense 5′ ATTAGGCAGAGGTGAAAAAG 3′ (nt 1839 to 1820). The amplification conditions were: an initial denaturation step at 94 °C for 5 min, then 94 °C for 30 s, 55 °C for 30 s, 72 °C for 30 s for 36 cycles and a final extension at 72 °C for 5 min. The PCR products were inserted into the pGEM-T Easy vector (Promega, Madison, WI, USA). The genes were excised from the plasmid with EcoR1 restriction enzyme (Promega, Madison, WI, USA) and subcloned into the mammalian expression vector pcDNA3.1 (Invitogen, Carlsbad, CA, USA) to generate the sgtF1b HBx and sgtF4 HBx plasmids. The sequence and correct orientation of the X gene was confirmed by sequencing analysis. The GenBank accession numbers for the HBx producing plasmids were: MK808039 and MK808040.

### 2.4. Transient Transfection 

For transfections, a full-length linear HBV DNA (nt 1820-1820) with sticky ends was used.

As previously described, linear HBV monomers were excised from the plasmids by restriction enzyme digestion with 5 U of BspQI (New England Biolabs, Beverly, MA, USA) at 50 °C. The 3.2 kb fragments were gel purified with PureLink Quick Gel Extraction Kit (Invitrogen, Carlsbad, CA, USA), according to the manufacturer’s instructions and the DNA was quantified spectrophotometrically [20].

For transfections, cells were seeded in 6 or 24 well plates and grown to 60–70% confluence. Transfections were carried out using X-tremeGene 9 transfection reagent (Roche, Mannheim, Germany), according to the manufacturer’s recommendations. Transfection with a linear full-length HBV genome derivative from the pCH-9/3091 POL minus plasmid was used as control for evaluating the amount of remaining input DNA. For trans-complementation assays, cells were co-transfected with sgtF1b X(-) or sgtF4 X(-) genomes and sgtF1b HBx or sgtF4 HBx expression plasmids, respectively. Cells were maintained at 37 °C in 5% CO_2_ atmosphere. After 6 h incubation, medium was replaced, and cultures were incubated for 72 or 96 h. In order to eliminate HBV DNA input, cell culture medium was collected every 24 h, cells were washed six times with PBS, and fresh medium was added.

### 2.5. Transfection Efficiency

In all transfection experiments, 0.05 µg of the luciferase reporter vector pGL4.13 [luc2/SV40] (Promega, Madison, WI, USA) was co-transfected with the full-length linear HBV genomes as transfection efficiency control. To evaluate the light emission produced by the luciferase activity, 20 µL of cell lysates were mixed with the commercial reagent Luciferase Assay System (Promega, Madison, WI, USA), according to the manufacturer’s recommendations, and relative light units (RLU) were detected. Results were expressed per relative light units.

### 2.6. Analysis of Intracellular HBV DNA

HBV replicative intermediates were measured by Southern blot. Ninety-six hours post-transfection, cells were treated with lysis buffer (50 mM Tris/HCl pH 7.5; 100 mM NaCl; 1 mM EDTA; 0.5% NP40) and centrifuged 10 min at 4000 g. This is a weak lysis buffer which does not break cell nucleus; thus, the observed replication intermediaries will not contain cccDNA. After centrifugation, 20 µl of the cell lysate supernatant was used for luciferase detection assay and the rest was treated with 0.5 mg/ml proteinase K (Invitrogen, Carlsbad, CA, USA) at 65 °C for 3 h. Nucleic acids were purified by phenol-chloroform extraction and ethanol precipitation in the presence of 20 µg of dextran.

DNA isolated from cell lysates was separated on a 1% agarose gel and blotted onto a nylon positive membrane (Roche, Mannheim, Germany). The transferred membrane was immobilized by an ultraviolet crosslinker and hybridized with a subgenomic digoxigenin (DIG)-labelled probe (Roche, Mannheim, Germany). The hybridization signals were detected on an X-ray film using an enzyme-linked immunoassay (DIG Luminescent Detection Kit; Roche, Mannheim, Germany) and were quantified with the ImageJ software (Wayne Rasband, NIH, USA).

### 2.7. Analysis of Extracellular HBV DNA

Viral progeny was quantified by Real-time PCR. Ninety-six hours post-transfection, cell culture supernatants were harvested and clarified by centrifugation at 3000 g for 10 min. Supernatants were treated with lysis buffer (50 mM Tris-HCl pH 7.5; 1 mM EDTA; 1% SDS; 0.5 mg/mL proteinase K (Invitrogen, USA)) and incubated at 65 °C for 3h. Nucleic acids were purified by phenol-chloroform extraction and ethanol precipitation in the presence of 20 µg of dextran.

Quantitative Real-time PCR (qPCR) was performed in a 7500 Real-Time PCR System (Applied Biosystems, Foster City, CA, USA) using Luna Universal qPCR Master Mix (2×; New England Biolabs, Beverly, MA, USA). The following primers were used for the amplification: sense 5′-ATGGAGACCACCGTGAACGC-3′ (nt 1608 to 1627) and antisense 5′-AGGCACAGCTTGGTGGCTTG-3′ (nt 1887 to 1868). The position of the primers amplifies almost exclusively the circular HBV DNA genome, reducing significantly the amplification of the input genome (linear HBV DNA) used for transfection. The cycling conditions were as follows: an initial denaturation step at 95 °C for 3 min, followed by 40 cycles of 95 °C for 15 s, 61 °C for 30 s and 72°C for 30 s. Serial dilutions of a HBV replication-competent plasmid (pCH-9/3091) over a range of 10^2^ to 10^7^ copies were used as quantification standards. A standard curve (copies/mL) was used for quantification. All samples were performed in triplicate.

### 2.8. Analysis of Pre-Genomic RNA

pgRNA was quantified by Real-time PCR. Total cellular RNA from transfected HuH-7 cells at 72 h post-transfection was extracted with TRIzol reagent (Invitrogen, Carlsbad, CA, USA). RNA samples were treated with RQ1 RNase-free DNase (Promega, Madison, WI, USA) at 37 °C for 1 h, to remove contaminating DNA. RNA concentration and purity were determined by spectrometry. One microgram RNA was reverse transcribed into cDNA with Random Hexamer Primers (Biodynamics, Buenos Aires, Argentina) using M-MLV reverse transcriptase (Promega, Madison, WI, USA). Each cDNA was quantified by Real-time PCR using Universal qPCR Master Mix (2×; New England Biolabs, Beverly, MA, USA). The specific primers for pgRNA were: sense 5′-TTAATGACTTTGGCTTCCTGGG-3′ (nt 2093 to 2113) and antisense 5′-GAACTGTTTCTCTTCCAAAAGTAAG-3′ (nt 2247 to 2222); and for β-Actin were: sense 5′-CCCACACGGTGCCCATCTAT-3′ and antisense 5′-CCACGCTCCGTGAGGATCTTC-3′ [29]. Amplification of β-Actin cDNA was used to normalize the RNA samples.

### 2.9. Statistical Analysis

All experiments were independently performed three times. Statistical significance was determined using a two-tailed Student *t* test or one-way analysis of variance (ANOVA) followed by *post hoc* Bonferroni test when applicable. A value of *p* < 0.05 was considered to be statistically significant. Results were expressed as mean ± standard deviation. All analyses were performed using GraphPad Prism 8 software (GraphPad Software, San Diego, CA, USA, USA).

## 3. Results

### 3.1. HBx Modulates Wild Type Levels of sgtF1b and sgtF4 HBV Replication in HuH-7 cells

**Figure 2:**
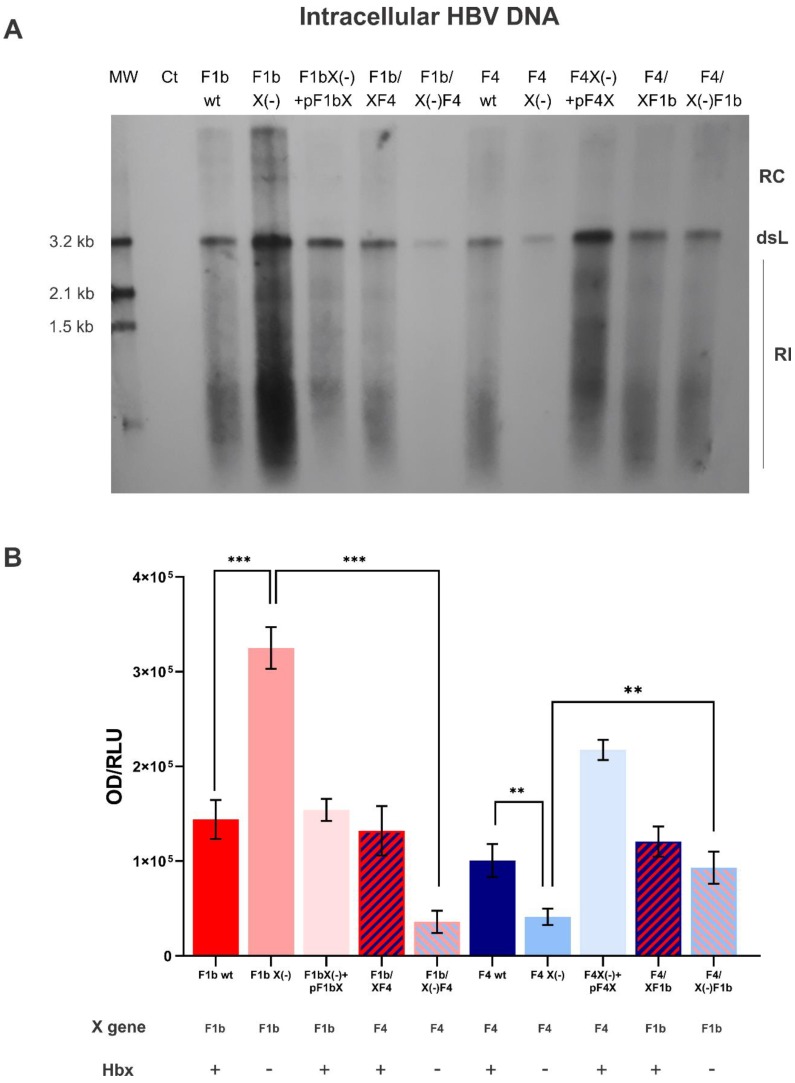
Analysis of intracellular HBV DNA of sgtF1b and sgtF4 variants. HuH-7 cells were transfected with linear full-length HBV genomes of pCH-9/3091 POL minus plasmid (Ct), F1b wt, F1b X(-), F1b X(-)+pF1bX, F1b/XF4, F1b/X(-)F4, F4 wt, F4 X(-), F4 X(-)+pF4X, F4/XF1b and F4/X(-)F1b variants. Ninety-six hours post-transfection, cells were harvest and the amount of HBV DNA replicative intermediates was assessed by Southern blot analysis (**A**). The DNA input control (Ct) was not visible, which shows no input interference in the Southern blot analysis. RC: HBV relaxed circular DNA; dsL: HBV double-stranded linear DNA; RI: HBV replicative intermediates. (**B**) Relative intensity of the bands was quantified using ImageJ software. Results were expressed in relation to luciferase relative light units (RLU). Shown values represent the mean ± standard deviation of three independent experiments. ** *p* < 0.005 and *** *p* < 0.0001.

**Figure 3:**
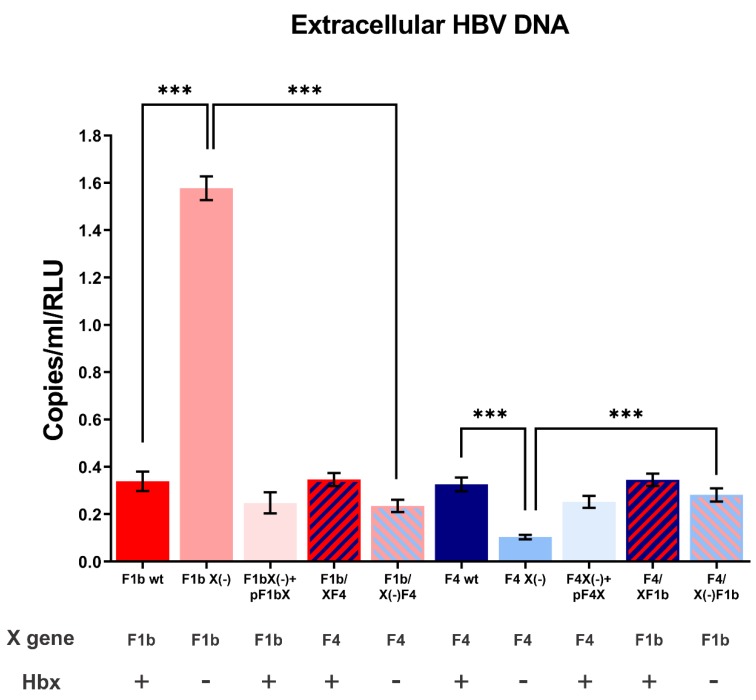
Analysis of extracellular HBV DNA of sgtF1b and sgtF4 variants. HuH-7 cells were transfected with linear full-length HBV genomes of F1b wt, sgtF1b X(-), F1b X(-)+pF1bX, F1b/XF4, F1b/X(-)F4, F4 wt, F4 X(-), F4 X(-)+pF4X, F4/XF1b and F4/X(-)F1b variants. Ninety-six hours post-transfection, cell supernatants were harvest and HBV DNA was extracted and quantified by real-time PCR. As a control, the remaining input DNA was also quantified (0.70 ± 0.03 copies/mL), and this value was subtracted from those obtained for the viral variants. Results were expressed in relation to luciferase relative light units (RLU). Shown values represent the mean ± standard deviation of three independent experiments. *** *p* < 0.0001.

To assess the role of HBx in sgtF1b and sgtF4 virus replication, HuH-7 cells were transfected with sgtF1b and sgtF4 wild type or knock-down X gene genomes (HBV X(-) genomes carry a stop codon in amino acid 8 of the X gene and do not express HBx). Ninety-six hours post-transfection cells and culture supernatants were harvested to examine the synthesis of HBV DNA intermediates by Southern blot and the production of extracellular HBV DNA by qPCR, respectively. Remarkably, as a consequence of HBx suppression, opposite effects in sgtF1b X(-) and sgtF4 X(-) replication levels were observed. For sgtF1b X(-), intracellular and extracellular HBV DNA levels significantly increased by 2.4 and 4.7 fold, respectively, when compared to sgtF1b wt. Meanwhile, sgtF4 X(-) intracellular and extracellular HBV DNA levels decreased in relation to sgtF4 wt, by 4.1 and 3.2 fold respectively (Figure 2 and Figure 3).

To verify that the effect observed was a consequence of the abrogation of HBx expression, we evaluated whether HBx ectopic expression restored sgtF1b X(-) and sgtF4 X(-) replication levels. Linear full-length sgtF1b X(-) and sgtF4 X(-) genomes were co-transfected with sgtF1b HBx and sgtF4 HBx expression plasmids, respectively. As shown in Figure 1 and Figure 2, co-transfection with a HBx expression plasmid restored replication of sgtF1b X(-) and sgtF4 X(-) genomes to sgtF1b wt and sgtF4 wt levels.

### 3.2. X Gene Sequence Regulates sgtF1b and sgtF4 HBV Replication

**Figure 4:**
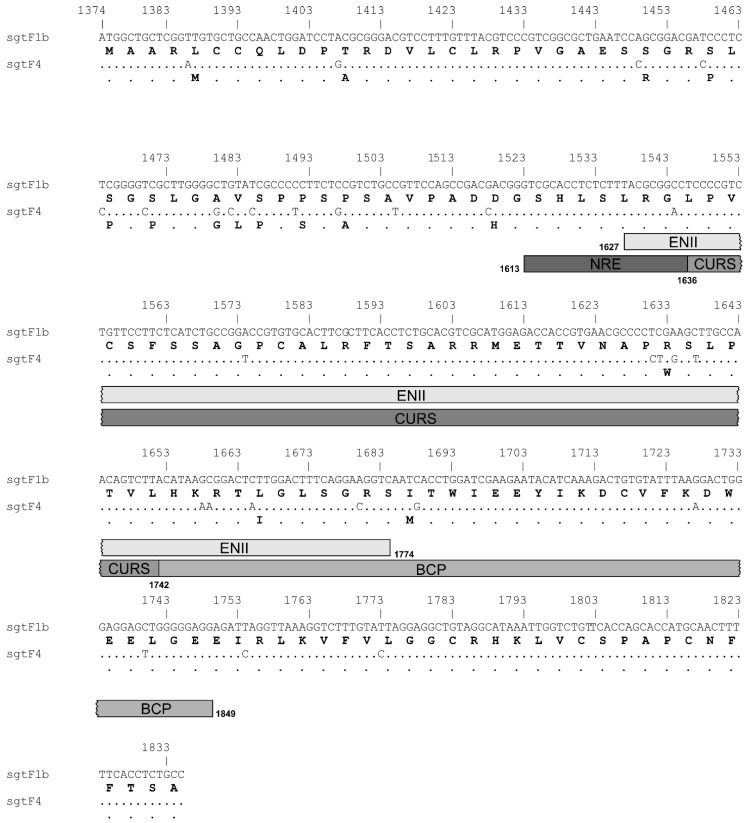
Nucleotide and amino acid alignment of sgtF1b and sgtF4 X gene/protein. The location of Enhancer II (ENII; nt 1627–1774) and Core Promoter (CP, 1575–1849), that overlap the X gene are shown. The CP comprises a negative regulatory element (NRE, nt 1613–1636), a core upstream regulatory sequence (CURS, nt 1636–1742) and the basal core promoter (BCP; nt 1743–1849).

The comparative analysis at nucleotide and amino acid level of prototypes of sgtF1b and sgtF4 X gene and protein, revealed 6% of divergence in the nucleotide sequences and 10% in the amino acid sequences (Figure 4).

To test whether the disparity observed in sgtF1b X(-) and sgtF4 X(-) replication was due to differences in the in the activity of HBx or in regulatory sites in the X gene of these subgenotypes, chimeric HBV genomes were designed.

On one hand, the X gene of sgtF1b and sgtF4 were swapped into sgtF4 and sgtF1b genomes, respectively. In this manner, sgtF4 genomes express sgtF1b HBx (F4/XF1b), and sgtF1b genomes express sgtF4 HBx (F1b/XF4). On the other hand, sgtF1b X(-) and sgtF4 X(-) genes, including the stop codon at amino acid 8, were swapped into sgtF4 and sgtF1b genomes, respectively. Thus, sgtF4 genomes include only the sequence of sgtF1b X gene (F4/X(-)F1b) and sgtF1b include only the sgtF4 X gene (F1b/X(-)F4).

HuH-7 cells were transfected with F1b/XF4, F1b/X(-)F4, F4/XF1b, and F4/X(-)F1b chimeric genomes. Intracellular HBV DNA intermediates and extracellular HBV DNA synthesis were analyzed by Southern blot and qPCR, respectively. No significant differences were observed in intracellular and extracellular HBV DNA levels between F1b/XF4 and sgtF1b wt, nor between F4/XF1b and sgtF4 wt viral variants (Figure 2 and Figure 3). These results support that HBx activity was independent of its subgenotype of origin.

In contrast, for F1b/X(-)F4 variant, intracellular and extracellular HBV DNA levels decreased 9 fold and 6.7 fold in relation to sgtF1bX(-), respectively. In fact, replication levels of F1b/X(-)F4 were similar to those observed for sgtF4 X(-) variant. Meanwhile, for F4/X(-)F1b variant, intracellular and extracellular HBV DNA levels increase by 2.3 and 2.7 fold in relation to sgtF4X(-), respectively (Figure 2 and Figure 3).

### 3.3. X Gene Sequence of sgtF1b and sgtF4 Modulates pgRNA Transcription

**Figure 5:**
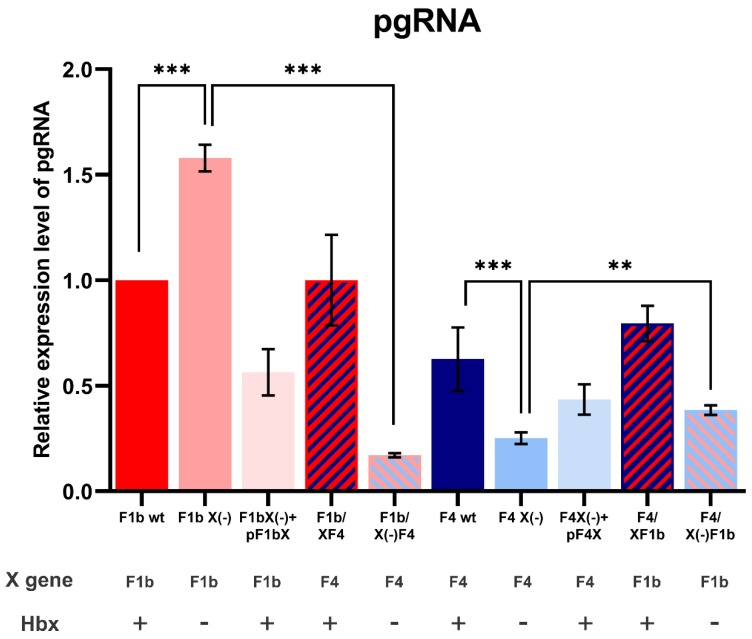
Analysis of pgRNA levels of sgtF1b and sgtF4 variants. HuH-7 cells were transfected with linear full-length HBV genomes F1b wt, F1b X(-), F1b X(-)+pF1bX, F1b/XF4, F1b/X(-)F4, F4 wt, F4 X(-), F4 X(-)+pF4X, F4/XF1b and F4/X(-)F1b variants. Seventy-two hours post-transfection, total RNA was extracted and pgRNA was quantified by RT-qPCR using specific primers. β-actin amplification was used to normalize each RNA sample. Shown values represent the mean ± standard deviation of three independent experiments. ** *p* < 0.005 and *** *p* < 0.0001.

To further investigate the effect of the X gene/protein on sgtF1b and sgtF4 viral replication, pgRNA levels were analyzed. HuH-7 cells were transfected with sgtF1b and sgtF4 wild type, HBx minus and chimeric genomes. Seventy-two hours post-transfection pgRNA levels were quantified by RT-qPCR. Interestedly, similar results as those obtained for intracellular and extracellular HBV DNA levels were observed (Figure 4). Transfection with sgtF1b X(-) significantly increased pgRNA levels in relation to sgtF1b wt, whereas, sgtF4 X(-) pgRNA levels significantly decreased compared to sgtF4 wt. Likewise, trans-complementation with sgtF1b HBx and sgtF4 HBx expression plasmids partially restore sgtF1b wt and sgtF4 wt pgRNA levels. Transfection with wild type chimeric genomes, F1b/XF4 and F4/XF1b, showed no significant differences in pgRNA levels in relation to sgtF1b wt and sgtF4wt, respectively. On the opposite, transfection with F1b/X(-)F4 decreased pgRNA levels in relation to sgtF1bX(-), meanwhile, for F4/X(-)F1b variant, pgRNA levels increase in relation to sgtF4X(-) (Figure 5).

Altogether these results show that, as was observed for DNA replication, the X gene sequence of sgtF1b and sgtF4 play a crucial role in pgRNA transcription and that HBx modulates pgRNA transcription toward wild type levels.

## 4. Discussion

In this study we report evidence of a differential modulation of HBV sgtF1b and sgtF4 transcription and replication by the X gene in HuH-7 cells. These subgenotypes, native from Latin America, present epidemiological as well as biological differences. It has been reported that sgtF1b possess a higher replication capacity and a major fitness compared to sgtF4 [20,21]. In addition, sgtF1b, the most prevalent in acute and HBeAg positive chronic infections in Argentina, has a delayed HBeAg seroconversion in comparison with genotypes/subgenotype A, F4 and D and, most importantly, it has been associated with a more severe course of chronic infection and HCC progression [18,19,22,24].

The X gene encodes HBx, a multifunctional regulatory protein with transcriptional transactivator activity on numerous cellular and viral promoters. Despite the large number of studies regarding its function, the precise role in HBV life cycle is controversial.

In the present study it has been shown that, even though HBx is not essential for viral replication, it would play a crucial role in modulating wild type sgtF1b and sgtF4 replication. These findings are consistent with several reports on the regulatory role of HBx in HBV replication [6,8,9,30]. In these works, HBx is described as an enhancer of viral replication, because the absence of HBx causes a drastic decrease in HBV replication levels; which can be restored after HBx ectopic expression. In the present work, this behavior was verified for sgtF4 X(-). Unexpectedly, sgtF1b X(-) behaved in the opposite way. Likewise, in both subgenotypes, HBx expression was able to rescue wild type replication levels. The results showed an antagonistic role of sgF1b and sgtF4 X gene or HBx.

It must be taken into account that previous studies analyzing the role of HBx in HBV replication has been carry out mainly in genotype D variants and to a lesser extent in genotypes A and B viruses. To our knowledge this is the first report to study the function of HBx in genotype F variants.

The up and down regulation of HBV DNA levels in sgtF1b X(-) and sgtF4 X(-), occurred in concomitance to pgRNA transcription levels, suggesting that the modulation of HBV DNA replication by HBx is the result of the regulation of HBV RNA transcription. These findings are consistent with several *in vitro* and *in vivo* studies performed in other genotypes [8,11,30].

Altogether, these results suggest that sgtF1b and sgtF4 viruses exploit different pathways to regulate its transcription and replication, which eventually lead to a common purpose, to modulate wild type replication levels of sgtF1b and sgtF4 viruses. This would imply a remarkable finding from the evolutionary aspect of the biology of the virus.

The comparative analysis of sgtF1b and sgtF4 X gene and protein sequences revealed differences at nucleotide and amino acid levels. In order to explained the disparity observed in sgtF1b X(-) and sgtF4 X(-) transcription and replication, two hypothesis may be proposed. On one hand, differences at protein level, in the activity of HBx. This would imply that sgtF1b HBx has an inhibitory role in HBV transcription and replication, whereas, sgtF4 HBx plays an enhancer role. To test the hypothesis, chimeric genomes in which sgtF4 virus express F1b HBx (F4/XF1b) and sgtF1b variants express F4 HBx (F1b/XF4) were designed. No differences were observed in HBV DNA and RNA levels between chimeric genomes (F1b/XF4 and F4/XF1b) and sgtF1b wt and sgtF4 wt viral variants. This result supports that HBx modulates the differences in transcription and replication regardless of the subgenotype of origin.

On the other hand, differences at genetic level, in the nucleotide sequence of the X gene. To test this hypothesis, chimeric genomes in which sgtF4 virus include only the sequence of sgtF1b X gene (F4/X(-)F1b), and sgtF1b virus include only the sgtF4 X gene (F1b/X(-)F4) were design. Interestedly, F1b/X(-)F4 DNA and RNA levels decreased in relation to sgtF1bX(-), and F4/X(-)F1b DNA and RNA levels increased in relation to sgtF4X(-). This result would indicate that the X gene sequence is the responsible for the disparity observed in HBV DNA and RNA levels in sgtF1b X(-) and sgtF4 X(-) variants.

HBV transcription is regulated by four promoters (core, preS1, preS2 and X) and two enhancers, I and II (EN I and EN II). In particular, pgRNA synthesis is regulated by the core promoter (nt 1575-1849). The core promoter consists of the basal core promoter (BCP; nt 1743-1849) and the upper regulatory region (URR, nt 1613-1742), the latter containing both positive (CURS, nt 1636-1742) and negative (NRE, nt 1613-1636) regulatory elements that modulate promoter activity [31,32]. In addition, the activity of the core promoter is further augmented by EN II. EN II (nt 1627-1774) is located upstream of the core promoter and increases preferentially the transcription of the pc/pg mRNAs [33,34]. It has been shown that several transcriptional factors bind to regulatory sequence elements of the core promoter and EN II to regulate pgRNA transcription [35].

It must be taken into account that regulatory sequence elements that modulate pgRNA transcription overlap with the X gene (Figure 3). Among the nucleotide divergence observed between sgtF1b and sgtF4, 13 of these changes (10 synonymous and 3 non-synonymous substitutions) were present in EN II and core promoter regions (including BCP and URR). The disparity in pgRNA levels in sgtF1b X (-) and sgtF4 X (-) variants might be due to a differential regulation in the activity of the core promoter and EN II and/or an uneven interaction of transcription factors with these regulatory regions. In this regard, this hypothesis agrees with a previous study reporting that the lower replication capacity of sgtC2 in relation to sgtB2 was associated to a weaker EN II/core promoter activity in Huh-7 cells. Accordingly, this weaker EN II/core promoter activity was partly attributable to 4 nucleotide positions displaying genotype-specific variability [36].

As mentioned, several reports have associated HBV genotypes and subgenotypes with differences in clinical and virological characteristics, such as replication capacity, HBeAg seroconvertion rate and disease progression [37,38,39]. The findings obtained in this work contribute to support the biological differences observed among sgtF1b and sgtF4.

In summary, the in-deep characterization of the X gene and the encoded X protein contribute to understand its biological role in two subgenotypes scarcely studied so far. The antagonistic role of the X gene in sgtF1b and sgtF4 reveals an unreported underlying evolutionary mechanism aimed to modulate HBV replication.
